# Comparative study on clinical efficacy of different methods for the treatment of intramural aortic hematoma

**DOI:** 10.1038/s41598-021-91151-0

**Published:** 2021-06-03

**Authors:** Junfu Luo, Wenpeng Zhao, Jiasheng Xu, Rui Zou, Kaihua Zhang, Yanhua Wan, Shasha Wan, Riwei Wang, Qingfu Zeng

**Affiliations:** 1 Department of Vascular Surgery, The Second Affiliated Hospital of Nanchang University, No. 1 Minde Road, Nanchang, 330006 Jiangxi Province China; 2 Department of General Surgery, The Jiujiang Affiliated Hospital of Nanchang University, No. 1 Minde Road, Nanchang, 330006 Jiangxi Province China; 3 Department of Emergency, The Second Affiliated Hospital of Nanchang University, No. 1 Minde Road, Nanchang, 330006 Jiangxi Province China

**Keywords:** Interventional cardiology, Outcomes research

## Abstract

To explore the difference of curative effect between different treatment modalities, in order to provide reference for the treatment of aortic intramural hematoma (IMH). 168 patients with aortic intramural hematoma diagnosed and treated from January 2010 to July 2020 were selected in the Second Affiliated Hospital of Nanchang University. Among them, 48 patients were diagnosed with Stanford A aortic intramural hematoma and 120 were diagnosed with Stanford B aortic intramural hematoma. According to the therapeutic methods, patients were divided into conservative treatment group and endovascular treatment group (TEVAR). For endovascular treatment group, according to the different timing of surgery, can be divided into acute phase group (onset within 72 h) and non-acute phase group (time of onset > 72 h).The clinical data and follow-up data were collected and analyzed by variance analysis and χ^2^ test. There were 168 patients diagnosed with aortic intramural hematoma 39 of them were (81.25%) Stanford A aortic intramural hematoma patients with pleural or pericardial effusion. For patient with Stanford A aortic intramural hematoma, endovascular treatment was performed in 15 patients (31.2%), and 33 cases (68.8%) for conservative treatment. The average follow-up (24.9 ± 13.9) was months. There were 120 patients with Stanford type B aortic intramural hematoma (71.4%), 60 patients received endovascular treatment (50%), and 60 patients (50%) received conservative treatment, with an average follow-up of (27.8 ± 14.6) months. For Stanford A type aortic intramural hematoma patients when the maximum aortic diameter ≥ 50 mm or hematoma thickness ≥ 11 mm, with high morbidity and mortality, positive endovascular treatment can reduce complications and death. For patients with Stanford type B aortic intramural hematoma, when the maximum aortic diameter ≥ 40 mm or hematoma thickness ≥ 10 mm, with high morbidity and mortality, positive endovascular treatment can reduce complications and death. Both Stanford type A and B aortic intramural hematoma patients could benefit from the endovascular treatment when the initial maximum aortic diameter is ≥ 50 mm or the hematoma thickness is ≥ 11 mm.

## Introduction

Acute aortic syndrome (AAS)^1–10^ is used to describe a life-threatening aortic disease with similar clinical symptoms but different population morbidity and pathophysiological changes, including aortic dissection (AD), penetrating aortic ulcer (PAU) and intramural hematoma (IMH). IMH indicates blood confined to the middle layer of the aorta, without intimal tears, and no blood flow to the aortic lumen^11^. The reason for its production is often thought to be rupture of the media nutrient artery or bleeding of intra-atherosclerotic plaque^12^. Its incidence accounts for about 10–30% of AAS^13–15^. It has been reported in the literature^16–18^ that about 10% of IMH could be absorbed and fully recover, while 8–78% of patients could progress to typical aortic dissection.

In recent years, with the advancement of imaging technology, the diagnosis and identification of IMH has been significantly improved, but the consensus on the best management strategy for this disease has not yet been established^19,20^. For the standard treatment plan of IMH, there is still controversy. According to the location and complications of aortic lesions, either drug therapy, traditional open surgery, or endovascular repair can be selected. The goal of drug therapy is to reduce the rate of systolic blood pressure and intraventricular pressure change (dP/dt), thereby reducing the stress on the aortic wall^21^. The anatomical goal of traditional surgery is to remove the aortic lesions and replace the graft, and reconstruct the layers in the distal anastomosis to block the blood flow to the false lumen^11^. Thoracic endovascular aortic repair (TEVAR) is rapidly promoted clinically with its minimally invasive and safe advantages, especially for elderly patients with poor general condition and intolerance to traditional surgery. It has been reported^18^ that the hospital mortality rate for Stanford type A IMH was 39%. Moreover, the mortality rate of surgical treatment and drug-only treatment was 44% and 33%, respectively. While the mortality rate for Stanford type B IMH hospitalization was 8%. Because of its high risk of rupture or progression to AD, especially in Stanford type A IMH patients, Stanford A-type IMH patients with or without ulcers have been surgically treated over the past decade, with a 30-day postoperative mortality report ranging from 10 to 50%, and the risk of observation and medication was much higher than surgery^22–24^. In addition, other investigators advocate conservative treatment of type A IMH and report favorable outcomes such as no typical dissection, and pericardial tamponade after conservative treatment^25–28^. Song et al.^27^ recommend that for IMH patients, medical conservative treatment could be performed first, followed by close imaging follow-up, and surgical treatment should be performed when complications occurred. Relative to the risk factors for IMH patients, some researchers suggested^28^ that maximal aortic hematoma thickness and enlarged aortic diameter were significant factors leading to complications or death in patients. Besides, some researchers believed that^18,29^ for IMH involving the ascending aorta, surgical treatment was advocated. For IMH involving the descending aorta, medication was the best choice. Recurrent or persistent pain, maximum aortic diameter > 50 mm or hematoma thickness > 11 mm, chest or pericardial effusion were regarded as indications for surgical intervention. Others suggested that^27,30^ the patient's maximum aortic diameter > 50-55 mm, hematoma thickness > 10-16 mm were regarded as risk factors for death, rupture and progression to the dissection.

The present study aimed to evaluating different treatments of IMH through conducting a single-centre, retrospective study in our hospital.

## Materials and methods

### Research content

#### Research objects and sources

**Figure 1:**
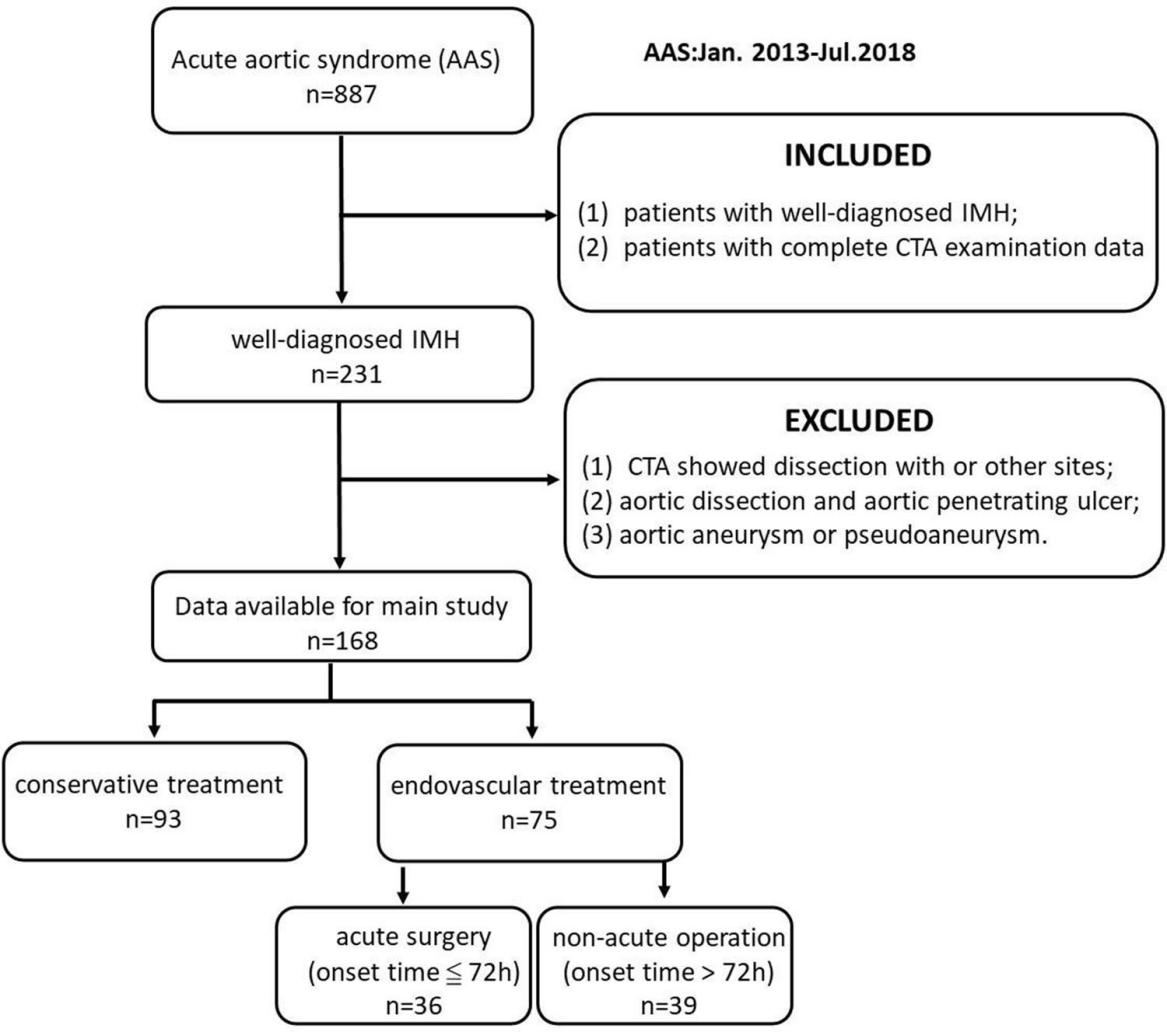
Flowchart of the study experimental object selection.

We retrospectively collected data from 168 patients who met the inclusion criteria and exclusion criteria from the Second affiliated hospital of Nanchang University in China from January 2013 to July 2018 (Fig. 1).

*Inclusion criteria* (1) patients with IMH; (2) patients with complete CTA examination data.

*Exclusion criteria* (1) admission CTA showed dissection with or other sites; (2) aortic dissection and aortic penetrating ulcer; (3) aortic aneurysm or pseudoaneurysm.

#### Grouping

The included patients were diagnosed by CTA after admission. According to the different treatment methods, patients were divided into conservative treatment group and TEVAR group. For the patients in the TEVAR group, according to the timing of surgery, they were divided into acute surgery group (onset time ≦ 72 h) and non-acute operation group (onset time > 72 h). Criteria for selection of treatment: Conservative treatment: 1. simple hypertension; 2. blood pressure less than 130 mmHg after drug control; 3. no obvious symptoms such as severe pain. Surgical treatment: 1. No relief of pain or recurrence of pain under active medical treatment; 2. Rapid local expansion of aorta; 3. Leakage or rupture risk, especially on the basis of original aortic aneurysm; 4. Compression of large branch vessels; 5. Original connective tissue; 6. Local ulcer or ulcer like protrusion lesions.

#### General treatment principles

First of all, in the acute phase of conservative drug treatment, the blood pressure, heart rate, pain should be strictly controlled. After passing the acute stage, CTA was reexamined. If the hematoma was absorbed and there was no clear break, conservative treatment was recommended. If the hematoma is enlarged and there is a clear endometrial break, TEVAR is performed. In TEVAR treatment, the proximal end of the stent should be anchored to the normal artery, the diameter of the stent should be accurately measured, and the low oversize stent should be selected to reduce the probability of stent related new break. The patients with conservative treatment and stent implantation were included in the close follow-up plan to strictly control blood pressure and adjust lifestyle. After 1 month, 3 months, 12 months, follow-up CTA every year, once the patient had complications, leakage and other abnormalities, timely symptomatic treatment.

### Research methods

#### CTA application and related data collection

All patients underwent CTA examination after admission. The vascular surgeon and interventional radiologist who were not aware of the contents of this study were jointly diagnosed on the CT workstation, and the software was used to measure the maximum aortic diameter and intramural hematoma thickness of the IMH patients (maximum aortic diameter: the longest diameter measured by the maximum cross-sectional area of the aorta; the thickness of the hematoma: the thickness measured at the maximum location of the aortic hematoma).

#### Conservative treatment

All patients were closely monitored to control blood pressure, heart rate, and analgesia was performed. After discharge, the patient continued to control blood pressure and heart rate, and regularly used a series of imaging examinations to monitor, such as CTA. The first review image was taken before discharge, and every 6 months. If the two review images showed stable lesions, then the follow-up period should be extended to 1 year.

#### Thoracic endovascular aortic repair (TEVAR)

All surgical patients were closely monitored and actively treated with medicine after admission. On this basis, surgical treatment was performed. Surgical indications^31^: 1. a type of aortic intramural hematoma patients were recommended for emergency surgery; 2. complex B type aortic intramural hematoma patients were recommended for thoracic aortic endovascular repair; 3. complex B type aorta for intramural hematoma patients. In this study, due to individual differences and the will of the patient’s family, some patients were treated conservatively or intracavitally.

Surgical method: The patient was placed in the supine position. Routine disinfection and draping were performed, and local anesthesia or general anesthesia was taken. The right/left inguinal oblique incision was performed to expose the common femoral artery. Then, the surgeon preseted the blocking band to block, and punctured the right/left common femoral artery under direct vision (or preseted Proglide vascular closure device after direct percutaneous puncture of the right/left common femoral artery according to Seldinger technique) and placed it into the 12F vascular sheath. Then, the surgeon placed it into the 5F gold-labeled Pigtail catheter through the guide sheath in the abdominal aorta and performed angiography of ascending aorta (speed 20 ml/s, total 30 ml, pressure 900 psi). According to the angiographic results, the relevant data were measured again, and the anatomical relationship and structural morphology of the lesion and the branch vessels were determined. The stent graft system was selected based on the preoperative CTA measurement results. The left upper extremity was used to puncture the brachial artery/radial artery (or the left upper arm/left neck incision exposed the left iliac artery/carotid artery) to place the 4F vascular sheath. The guide sheath was placed into the 4F pigtail catheter to the ascending aorta. Then, the surgeon exchanged superhard guide wire through the guide sheath from right/left femoral artery (0.035 inches from Lunderquist COOK company), exited from the guide sheath, put it into the stent delivery system along the guide wire, and accurately released the stent through attaching closely to the left subclavian artery open distal edge (e.g., proximal anchoring zone < 15 mm, the main body of the stent was placed close to the distal edge of the left common carotid artery/cephalic artery opening and released, placed the chimney stent from the left subclavian artery/common carotid artery or made a window at the original location). Next, the surgeon exited the coated stent delivery system, performed imaging evaluation again, and determined the presence of various complications. If there were complications which must be dealt with, the surgeon took corresponding measures in a timely manner. Finally, the blood vessel and the surgical wound were sutured, or the puncture site was sutured with a Proglide vascular suturing device. The puncture site and the puncture place were partially pressure-wrapped. In addition, intraoperative patients needed heparinization, and the systolic blood pressure of the patient was controlled to about 100 mmhg before the stent was released. The life signs of the patient were closely monitored during the operation.

#### Clinical data collection

The patient’s general clinical data include name, gender, age, symptoms and signs, smoking history, admission blood pressure, heart rate, blood lipids, conditions of combined underlying disease, diagnosis, time from onset to surgery, time of hospital stay, etc. The CTA data included whether or not the pericardial effusion or pleural effusion was combined and the initial maximum aortic diameter and hematoma thickness of the IMH patient. The conditions of surgical treatment include perioperative mortality, surgical success rate, and complications. The follow-up was mainly conducted by outpatient, inpatient CTA or telephone. The follow-up data included disease progression, complications and secondary intervention, IMH-related mortality and all-cause mortality.

### Statistical analysis

Statistical analysis was performed using SPSS 21.0 statistical software. The measurement data were expressed as mean and standard deviation (x ± S), and the count data was expressed by the number of cases and percentages. The comparison of measurement data was performed by Kolomogorov–Smirhov Z test. Χ^2^ test was used; non-parametric test was used for comparison between groups, and the difference was statistically significant at P < 0.05.

### Ethical approval

The study was approved by the medical ethics committee of the Second Affiliated Hospital of Nanchang University. All methods were performed in accordance with the relevant guidelines and regulations.

### Informed consent

All patients involved in the study signed the informed consent voluntarily. Informed consent of all dead patients involved in the study had been obtained from their legal guardians. All experimental protocols were approved by the Ethics Committee of the Second Affiliated Hospital of Nanchang University.

## Results

### IMH patients with clinical data and efficacy

#### Clinical data of IMH patients

There were 168 patients with IMH, 156 patients (92.9%) had symptoms on admission, with aortic pain, and the pain was associated with the lesion. 138 (82.1%) patients had a history of hypertension. 48 patients (28.6%) were diagnosed with Stanford type A, including 21 males and 27 females, aged from 46 to 85 years old, mean years old (65.6 ± 9.2). 43 (89.6%) Stanford A Patients with type IMH had pleural or pericardial effusions. 120 patients (71.4%) with Stanford type B patients. There were 78 males and 42 females, aged from 48 to 84 years old, with an average of (63.9 ± 10.4) years old. 42 (35%) patients with Stanford type B IMH had pleural or pericardial effusions. There was difference in whether there were chest or pericardial effusions between the two groups. The difference was statistically significant (*P* < 0.05). Patients with Stanford type A IMH had more pleural or pericardial effusions. See Table 1 For details.

**Table 1:** Comparison of risk factor of patients treated with two different methods (x ± S).

Risk items	Conservative treatment group (n = 93)	Endovascular treatment group (n = 75)	Total (n = 168)	*P*
Age (years old)	67.5 ± 8.2	66.1 ± 7.5	–	0.73
Total cholesterol (mmol/L)	3.95 ± 0.75	4.62 ± 0.78		0.62
Triglyceride (mmol/L)	1.20 ± 0.45	1.36 ± 0.84		0.65
High density lipoprotein (mmol/L)	1.32 ± 0.28	1.26 ± 0.30		0.75
Low density lipoprotein (mmol/L)	2.16 ± 0.45	2.65 ± 0.82		0.15
Symptoms (aortic pain) in admission [number (%)]	80 (88.89)	71 (91.03)	151 (89.88)	0.35
Male [number (%)]	41 (45.56)	37 (47.44)	78 (46.43)	0.55
Thoracic or pericardial effusion [number (%)]	43 (47.78)	32 (41.03)	75 (44.64)	0.20
History of smoking [number (%)]	21 (23.33)	19 (24.36)	40 (23.81)	0.50
Hypertension [number (%)]	73 (81.11)	63 (80.77)	136 (80.95)	0.95
Diabetes [number (%)]	2 (2.22)	2 (2.56)	4 (2.38)	–
Heart disease [number (%)]	5 (5.55)	4 (5.13)	9 (5.36)	0.99

#### Therapeutic effect of different treatments for patients with Stanford type A IMH

48 patients with Stanford type A IMH were admitted to our hospital (15 patients with the TEVAR, 9 patients with the acute surgery, 6 patients with non-acute surgery). Three CTA of TEVAR patients admitted to the hospital showed maximum aortic diameter ≥ 50 mm or hematoma thickness ≥ 11 mm in the acute surgery group with no postoperative leakage, hematoma reduction or absorption during follow-up. In non-acute surgery group, preoperative CTA showed progression of aortic dissection. Three case of type I had endoleak after operation, and after follow-up observation continued for 1 year, the endoleak disappeared. Other 3 cases had no endoleak and hematoma absorption. 33 patients in the conservative treatment group: 21 patients with CTA maximum aorta diameter ≥ 50 mm or hematoma thickness ≥ 11 mm, including 3 patients died of rupture during hospitalization, 3 patients died of rupture during follow-up, 12 patients progressed to B-type aortic dissection, and 3 cases without change when reviewed. 12 patients were with CTA with aortic diameter < 50 mm and hematoma thickness < 11 mm when admitted, and the hematoma was reduced or absorbed during follow-up. The total hospital mortality rate of patients in this group of conservative treatment was 9.1%.

#### Clinical factors, timing and methods of treatment on the efficacy of Stanford type A IMH patients

In Stanford type A IMH conservative treatment, the therapeutic effect is closely related to the maximum diameter of aorta and the thickness of hematoma. Patients with larger diameter and thicker hematoma are more likely to have complications or death. See Tables 2 and 3 for details.

**Table 2:** Comparison of risk factors in Stanford type A patients with or without complications.

Risk factor	Complications or death (19)	No complications (29)	total (n = 48)	*P*
Male	8 (42.11)	13 (44.83)	21 (43.75)	0.85
Female	11 (57.89)	16 (55.17)	27 (56.25)	0.91
Age	66.5 ± 3.5	64.5 ± 5.5	65.6 ± 9.2	0.55
Hypertension	14 (73.68)	96 (84.21)	39 (81.3)	0.20
Delayed diagnosis (h)	8.7 ± 2.5	9.4 ± 3.0	9.3 ± 2.7	0.4
History of smoking	6 (31.58)	3 (10.34)	9 (18.75)	0.012
Diabetes	1 (0.048)	1 (0.030)	2 (0.037)	–
Heart disease	1 (5.26)	2 (6.70)	3 (6.25)	–
Focal intimal rupture	5 (26.32)	3 (10.34)	8 (16.67)	0.020
Thoracic or pericardial effusion	20 (95.24)	23 (69.70)	43 (89.6)	0.035
Haematoma thickness ≥ 11 mm	8 (42.11)	1 (3.45)	9 (18.75)	0.001
Maximum aortic diameter ≥ 50 mm	10 (52.63)	2 (6.90)	12 (25.00)	0.001

**Table 3:** Comparison of risk factors in Stanford type B patients with or without complications.

Risk factor	Complications (35)	No complications (85)	Total (n = 120)	*P*
Male	20 (57.14)	58 (68.24)	78 (65)	0.7
Female	15 (42.86)	27 (31.76)	42 (35)	0.65
Age	65.1 ± 5.2	62.8 ± 6.3	63.9 ± 10.4	0.85
Hypertension	27 (77.14)	71 (83.53)	98 (81.67)	0.13
Delayed diagnosis (h)	8.5 ± 2.0	8.9 ± 3.5	8.8 ± 3.0	0.42
History of smoking	16 (45.71)	15 (17.65)	31 (25.83)	0.021
Diabetes	1 (2.86)	1 (1.18)	2	–
Heart disease	3 (8.57)	3 (3.53)	6	–
Focal intimal rupture	5 (14.28)	4 (4.71)	9	0.012
Thoracic or pericardial effusion	12 (34.29)	20 (23.53)	32	0.030
Haematoma thickness ≥ 11 mm	15 (42.86)	1 (1.18)	16	0.001
Maximum aortic diameter ≥ 50 mm	12 (34.29)	2 (2.35)	14	0.001

For patients with Stanford type A IMH TEVAR, the timing of treatment, aortic diameter, hematoma thickness for the treatment effect were not statistically significant. See Table 4 for details.

**Table 4:** Comparison of initial aortic diameter and hematoma thickness for conservative treatment of Stanford type A IMH.

Items	total	Complications* or death (n)	No complications (n)	Chi-square value	*P*
Maximum aortic diameter ≥ 50 mm or hematoma thickness ≥ 11 mm	21	18	3	75.4	2.32E−05
Maximum aortic diameter < 50 mm and hematoma thickness < 11 mm	12	0	12		< 0.01

*Progression of aortic dissection, pseudoaneurysm, rupture, visceral arterial perfusion, lower limb ischemia.

TEVAR is superior to conservative treatment for Stanford type A IMH patients with aortic diameter ≥ 50 mm or hematoma thickness ≥ 11 mm. See Tables 5 and 6 for details.

**Table 5:** Comparison of the therapeutic effect of acute and non-acute endovascular treatment for maximal aortic diameter ≥ 50 mm or hematoma thickness ≥ 11 mm.

Items	Type	Total	Complications* or death (n)	No complications (n)	Chi-square value	*P*
Maximum aortic diameter ≥ 50 mm or hematoma thickness ≥ 11 mm	Acute phase	9	0	9	18.8	0.17
	Non-acute phase	6	3	3		

*Progression of aortic dissection, pseudoaneurysm, rupture, visceral arterial perfusion, lower limb ischemia.

**Table 6:** Comparison of conservative and endovascular treatment for maximal aortic diameter ≥ 50 mm or hematoma thickness ≥ 11 mm.

Items	Type	Total	Complications* or death (n)	No complications (n)	Chi-square value	*P*
Maximum aortic diameter ≥ 50 mm or hematoma thickness ≥ 11 mm	Conservative treatment	21	18	3	51.8	0.03
	Endovascular treatment	15	3	12		< 0.05

*Progression of aortic dissection, pseudoaneurysm, rupture, visceral arterial perfusion, lower limb ischemia.

#### Curative effects of different treatments for patients with Stanford type B IMH

120 patients with Stanford type B IMH were admitted to our hospital, 60 patients received TEVAR, 33 patients received non-acute surgery, 60 patients received conservative treatment. The form of TEVAR group: 27 patients in the acute surgery group, 18 patients with CTA maximum aortic diameter ≥ 40 mm or hematoma thickness ≥ 10 mm. Type I endoleak occurred in 6 cases after operation.

In the remaining 12 patients, hematoma was reduced or absorbed during the follow-up period. In 9 patients admitted to the hospital, CTA showed that the maximum aorta diameter was < 40 mm and the hematoma thickness was < 10 mm. No intraoperative leakage was found, and the hematoma was reduced or absorbed during the follow-up period. 33 patients were in the non-acute surgery group, including 18 patients with CTA maximum aorta diameter ≥ 40 mm or hematoma thickness ≥ 10 mm. The preoperative CTA showed progression to type B aortic dissection, and there was no postoperative endoleak. The hematoma was reduced or absorbed during follow-up period. 60 patients were in the conservative treatment group: 30 patients with CTA maximum aorta diameter ≥ 40 mm or hematoma thickness ≥ 10 mm. There were 30 cases of patients whose CTA showed maximum aortic diameter < 40 mm and hematoma thickness < 10 mm after admitted to hospital, and the hematoma was reduced or absorbed during the follow-up period. The total hospital mortality rate of patients in this group of conservative treatment was 5.0%.

#### Effect of initial aortic diameter and hematoma thickness on the efficacy of Stanford type B IMH patients

In Stanford type B IMH conservative treatment, the therapeutic effect is closely related to the maximum diameter of aorta and the thickness of hematoma. Patients with larger diameter and thicker hematoma are more likely to have complications or death.

For patients with TEVAR, the timing of treatment, aortic diameter, hematoma thickness for the treatment effect were not statistically significant.

TEVAR is superior to conservative treatment for Stanford B IMH patients with aortic diameter ≥ 40 mm or hematoma thickness ≥ 10 mm. See Tables 7, 8, 9, 10 and 11 for details.

**Table 7:** Comparison of the maximum aortic diameter and hematoma thickness for the conservative treatment of Stanford B IMH.

Items	Total	Complications* or death (n)	No complications (n)	Chi-square value	*P*
Maximum aortic diameter ≥ 40 mm or hematoma thickness ≥ 10 mm	30	27	3	163.6	9.05E−12
Maximum aortic diameter < 40 mm and hematoma thickness < 10 mm	30	0	30		< 0.01

*Progression of aortic dissection, pseudoaneurysm, rupture, visceral arterial perfusion, lower limb ischemia.

**Table 8:** Comparison of the efficacy of acute and non-acute endovascular treatment in patients with Stanford type B IMH.

Items	Total (n)	Complications* or death (n)	No complications (n)	Chi-square value	*P*
Acute phase	27	6	21	2.72	0.40
Non-acute phase	33	0	33		

*Progression of aortic dissection, pseudoaneurysm, rupture, visceral arterial perfusion, lower limb ischemia.

**Table 9:** Comparison of the maximum aortic diameter and hematoma thickness for the treatment of Stanford B IMH.

Items	Total	Complications* or death (n)	No complications (n)	Chi-square value	*P*
Maximum aortic diameter ≥ 40 mm or hematoma thickness ≥ 10 mm	36	27	30	1.48	0.31
Maximum aortic diameter < 40 mm and hematoma thickness < 10 mm	24	0	24		

*Progression of aortic dissection, pseudoaneurysm, rupture, visceral arterial perfusion, lower limb ischemia.

**Table 10:** Comparison of conservative and endovascular treatment for maximal aortic diameter ≥ 40 mm or hematoma thickness ≥ 10 mm.

Items	Type	Total	Complications or death (n)	No complications (n)	Chi-square value	*P*
Maximum aortic diameter ≥ 40 mm or hematoma thickness ≥ 10 mm	Conservative treatment	30	27	3	117.3	7.62E−05
	Endovascular treatment	36	6	30		< 0.01

**Table 11:** Comparison of conservative and endovascular treatment for maximal aortic diameter < 40 mm and hematoma thickness < 10 mm.

Items	Type	Total	Complications* or death (n)	No complications (n)	*P*
Maximum aortic diameter < 40 mm and hematoma thickness < 10 mm	Conservative treatment	30	0	30	1
	Endovascular treatment	24	0	24	

*Progression of aortic dissection, pseudoaneurysm, rupture, visceral arterial perfusion, lower limb ischemia.

### Follow-up

**Figure 2:**
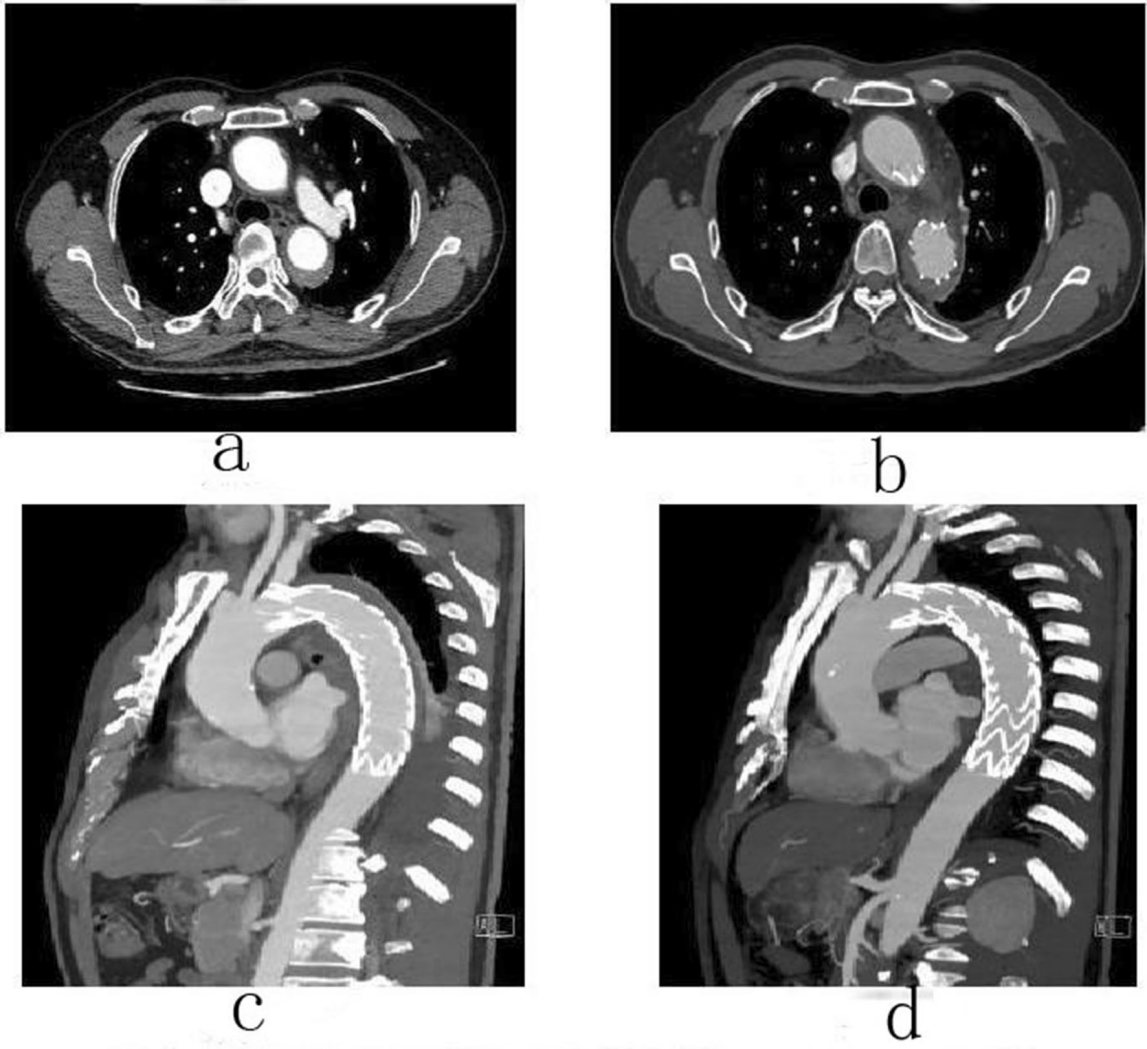
Male, 62 years old, admitted to hospital for “repetition of chest and back pain for 9 h”. Diagnosis: Standford A IMH TEVAR. CTA of the patient in different periods: (**a**) Admission; (**b**) 5 days after surgery; (**c**) 5 days after surgery; (**d**) 3 months after surgery.

**Figure 3:**
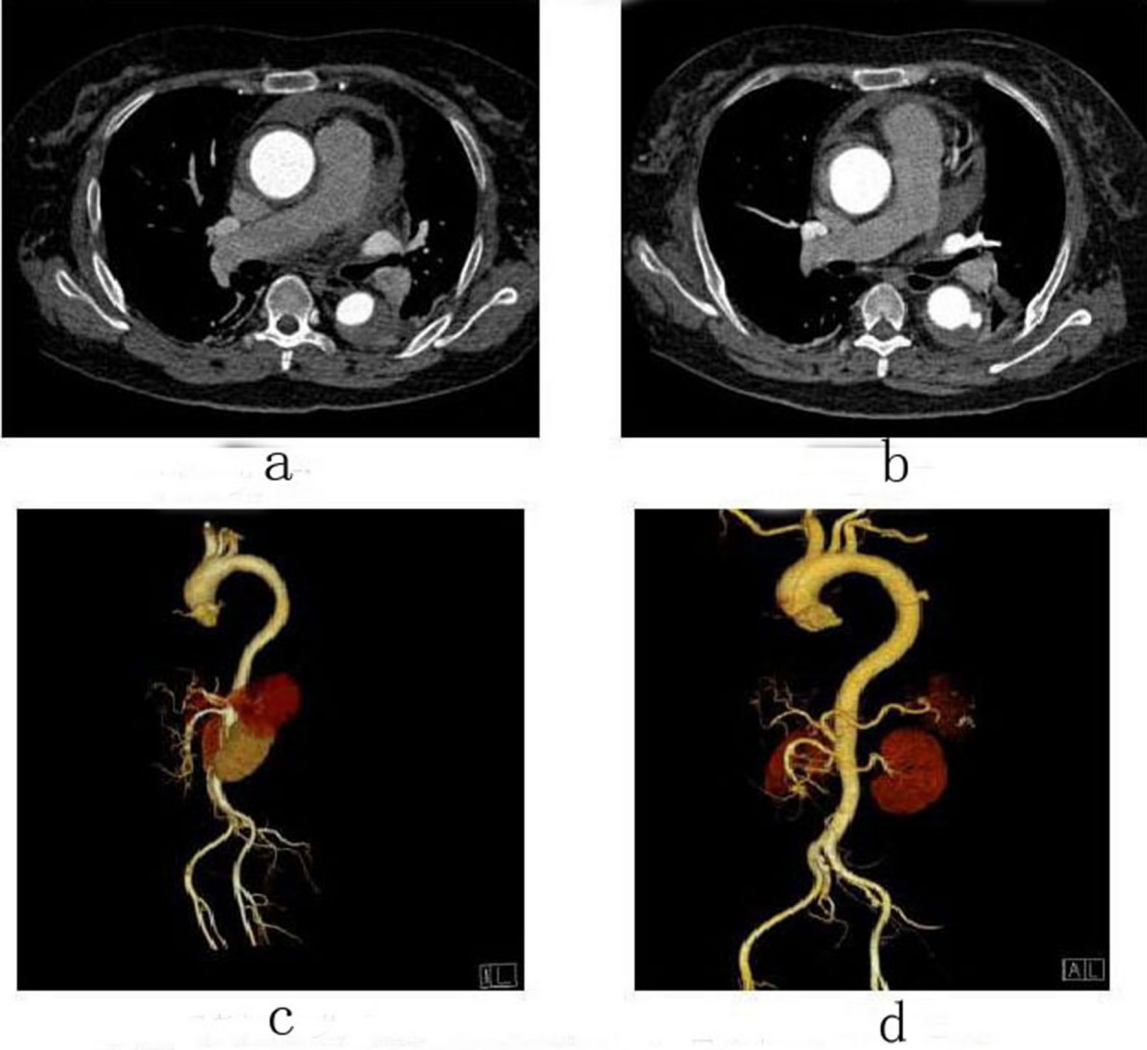
Female, 57 years old, admitted to hospital for “sudden pain in low back for 15 h”, Diagnosis: Stanford type A aortic dissection; treatment: IMH conservative treatment. CTA of the patient in different periods: (**a**) Admission; (**b**) 12 days after admission; (**c**) Admission; (**d**) 12 days after admission.

**Figure 4:**
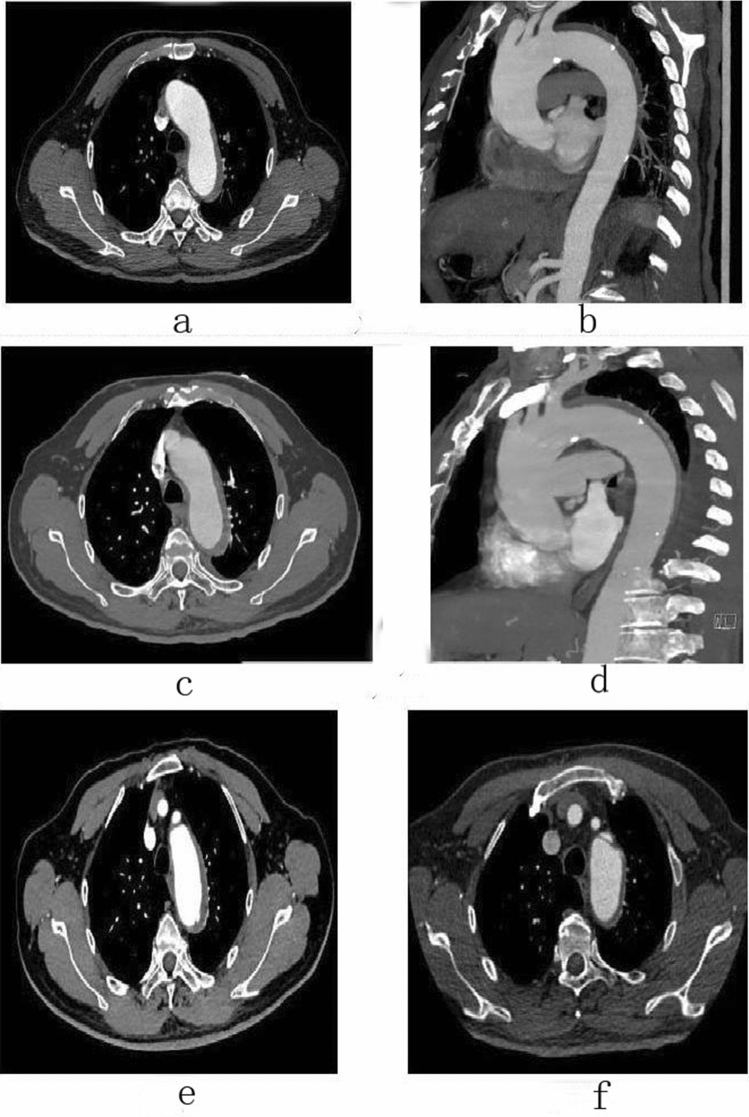
Male, 67 years old, admitted to hospital for “sudden pain in chest for 9 h”, Diagnosis: Stanford type B aortic dissection; Treatment: IMH conservative treatment. CTA of the patient in different periods: (**a**) Admission; (**b**) Admission; (**c**) 3 days after admission; (**d**) 3 days after admission; (**e**) 3 months after admission; (**f**) 11 months after admission.

**Figure 5:**
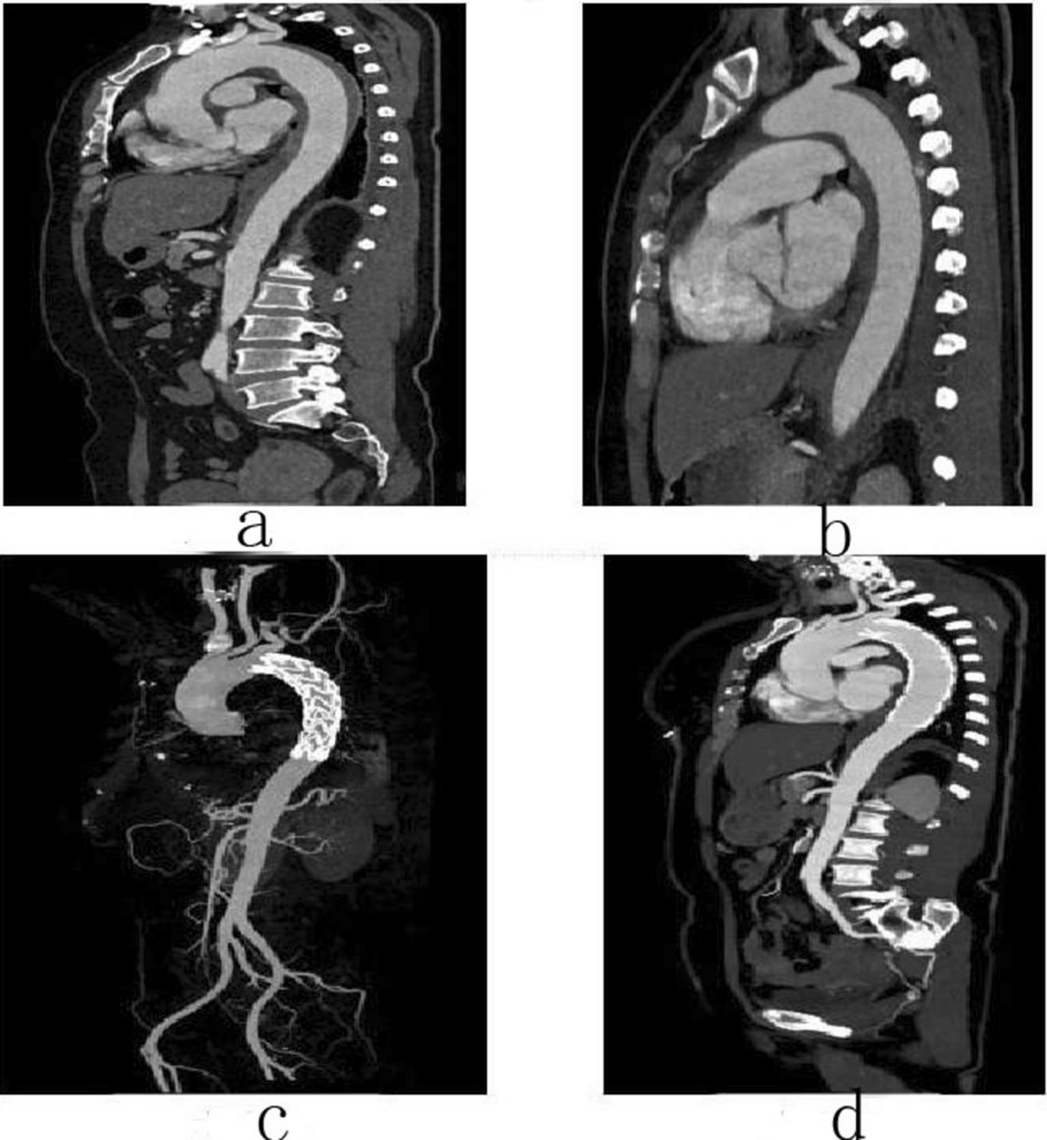
Female, 49 years old, admitted to hospital for “sudden tearing pain in low back for 48 h”, Diagnosis: Stanford type B aortic dissection; Treatment: IMH TEVAR treatment. CTA of the patient in different periods: (**a**) Admission; (**b**) 12 days after admission; (**c**) 2 days after surgery; (**d**) 4 months after surgery.

**Figure 6:**
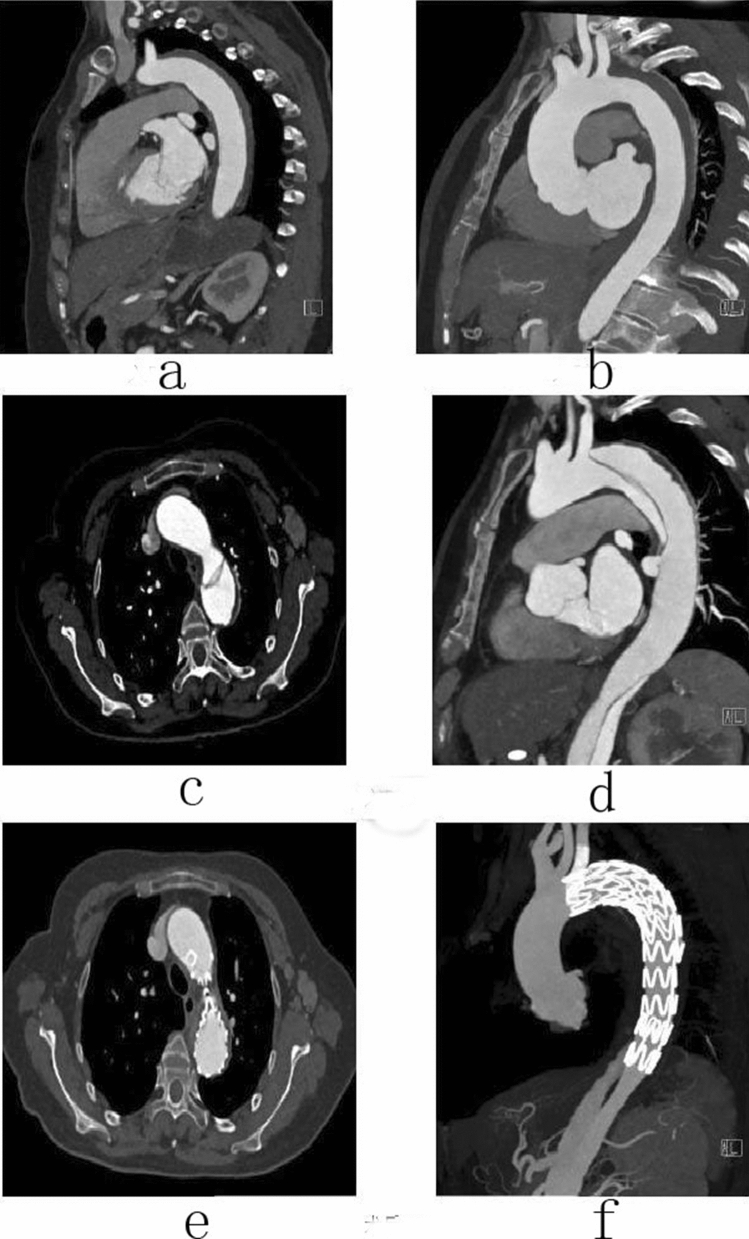
Female, 58 years old, admitted to hospital for “sudden pain in chest for 6 h”, Diagnosis: Stanford type B aortic dissection; Treatment: IMH TEVAR treatment. CTA of the patient in different periods: (**a**) Admission; (**b**) 5 days after admission; (**c**) 16 days after admission; (**d**) 16 days after admission; (**e**) 7 days after surgery; (**f**) 7 days after surgery.

A total of 168 patients were counted, and follow-up was performed mainly through outpatient, inpatient CTA or telephone. 144 patients had complete data. The overall follow-up rate was 85.7%, including 87.5% for type A and 85.0% for type B. The follow-up period ranged from 3 to 60 months. The mean follow-up time for conservative treatment group was 28.4 ± 12.5 months and 27.9 ± 14.5 months for TEVAR group. There was no significant difference between the two groups (*P* > 0.05). The follow-up data for conservative treatment patients included: incidence of complication, disease-related mortality, and all-cause mortality. The follow-up data for patients undergoing TEVAR included: endoleak condition, secondary intervention rate, incidence of complication, disease-related death rate, and all-cause mortality (Figs. 2, 3, 4, 5, 6). See Tables 12, 13 and 14 for details.

**Table 12:** Follow-up of conservative treatment patients [example (%)].

Items	Type A IMH group (n = 33)	Type B IMH group (n = 60)
Complications	12 (36.3)	21 (35.0)
Disease-related death	3 (9.1)	0 (0)
All cause death	3 (9.1)	3 (5.0)

**Table 13:** Follow-up of patients undergoing endovascular treatment [example (%)].

Items	Type A IMH group (n = 15)	Type B IMH group (n = 60)
Complications	3 (20.0)	6 (10.0)
Endoleak	3 (20.0)**	6 (10.0)**
Secondary intervention	0 (0)	0 (0)
Disease-related death	0 (0)	0 (0)
All cause death	0 (0)	0 (0)

**Are all type I internal leaks.

**Table 14:** Comparative analysis of the efficacy of different types of IMH patients.

Items	Total	Complications* or death (n)	No complications (n)	Chi-square value	*P*
Type A IMH group	48	21	27	1.38	0.13
Type B IMH group	120	33	87		

*Progression of aortic dissection, pseudoaneurysm, rupture, visceral arterial perfusion, lower limb ischemia.

By comparing the efficacy of different types of IMH patients, there was no significant difference in the incidence of complications or mortality between the two types of patients (*P* > 0.05), as shown in Table 14.

## Discussion

### Analysis of IMH clinical characteristics, treatment and efficacy

Pathologically, unlike typical aortic dissection, IMH does not have a decompression mechanism, but shows that non-communicating blood with intimal thickening or no echo. These differences also explain the risk of higher rupture or progression to AD in patients with IMH^16,17,31–33^, or possibility of hematoma regression and absorption^34^. The most common clinical manifestation is “aortic pain”. Chest pain often indicates that the lesion is located in the ascending aorta. Pain in the upper or lower back often indicates that the lesion is located in the descending aorta. Some patients have a combination of pericardium, pleural effusion or mediastinal effusion, and related studies^35^ believe that its occurrence was related to increased permeability of the aortic wall. In this study, IMH patients were more common in the elderly, 58.9% were male. 92.9% of the patients had symptoms on admission, which showed “aortic pain”, and the pain site was associated with the lesion. 82.1% had a history of hypertension; 90% patients with Stanford type A IMH had pleural effusion or pericardial effusion, and their formation mechanism and impact on patient prognosis needed to be further studied.

According to the onset time, the acute phase is within 14 days of onset, the subacute phase is from 14 to 60 days, and the chronic phase is after 60 days^5^. In terms of treatment, because IMH has a higher risk of rupture or progression to AD, early detection and active treatment are essential, and it can be treated with drugs, traditional surgery, TEVAR. Good drug therapy should be used throughout the whole process of the treatment. Since Dake et al.^36^ had successfully applied TEVAR to aortic dissection in 1994, TEVAR has been rapidly promoted clinically due to its minimally invasive and safe advantages, especially suitable for the elderly and those with poor systemic conditions who cannot tolerate traditional surgery. And it has a good near- and medium-term efficacy, the goal of which includes covering the vulnerable segment of the aorta, preventing the intimal tear, forming aortic dissection or rupture, and promoting complete thrombosis of the pseudo-cavity. TEVAR surgery was performed in all patients undergoing surgery in this study. There is still debate at home and abroad about the choice and criteria for IMH treatment options. For patients with Stanford type A IMH, because of the high mortality rate due to drug therapy alone, Evangelista et al.^37^ advocated active surgical treatment to avoid rupture or progression of the aorta dissection. For the treatment of Stanford type B IMH, studies have concluded that^38,39^ active surgical intervention was not the best treatment. Instead, it was recommended to initially take active medication, and interventional or open surgery was suitable for conditions of acute Stanford type B aortic dissection, such as persistent or recurrent pain, aortic dilation, anatomical progression, and end-organ perfusion syndrome. In this study, 15 patients (31.3%) underwent TEVAR in Stanford type A IMH patients, 3 patients occurred endoleak after surgery, and there was no death patients. Moreover, it might be relevant that all patients in this center underwent TEVAR and had high selectivity. 33 patients (68.7%) underwent conservative treatment and 6 patients (18.2%) died, which was lower than foreign reports. In Stanford type B IMH, 60 patients (50.0%) underwent TEVAR , 6 patients had endoleak after operation, and no patients died. 60 patients (68.7%) underwent conservative treatment, 3 patients (5.0%) died in hospital and 3 patients died during follow-up, and the total mortality rate was 10%. Another literature believes that^40,41^ the condition of maximum diameter of the aorta ≥ 50 mm, hematoma thickness ≥ 11 mm could predict adverse clinical prognosis, which could be used as a standard for clinical surgical intervention.

The study summarized and analyzed the clinical and follow-up data of IMH patients admitted to the center. The follow-up time ranged from 3 to 60 months. The average follow-up time was (28.2 ± 13.4) months, and the total follow-up rate was 85.7%. Among them, patients with Stanford type A IMH accounted for 87.5%, and patients with the Stanford Type B IMH accounted for 85.0%. The average follow-up time of Stanford type A IMH was (27.8 ± 13.9) months. Among the conservative patients, 12 patients developed AD, 6 patients died. Among the patients undergoing intraluminal treatment, there were 3 cases of type I leakage after surgery. All patients were admitted to the hospital with CTA showed a maximum aortic diameter ≥ 50 mm or a hematoma thickness ≥ 11 mm. 6 patients were with disease-related deaths and 6 patients were with all-cause death. The average follow-up time of Stanford B-type IMH was (28.3 ± 13.3) months. Among the conservative patients, 18 patients developed AD, 6 patients died, and 3 patients had left lower limb ischemia. Among patients undergoing intraluminal treatment, there were 6 patients with type I endoleak after operation, with maximal aortic diameter ≥ 40 mm or hematoma thickness ≥ 10 mm. 3 patients were with disease-related deaths and 6 patients were with all-cause death. Univariate analysis found that maximal aortic diameter and hematoma thickness were significant factors leading to complications and death. Stanford type A IMH patients were more prone to complications or death when the maximum aortic diameter was ≥ 50 mm or hematoma thickness was ≥ 11 mm, and the therapeutic effect of intraluminal treatment was better than conservative treatment. Patients with Stanford type B IMH had a greater maximum aortic diameter ≥ 40 mm or a hematoma thickness ≥ 10 mm, which was more prone to complications or death, and the efficacy of intraluminal treatment is better than conservative treatment. By comparing and analyzing the efficacy of different types of IMH patients, it was found that there was no significant difference between the two types, and there was a deviation from the domestic and international reports. Considering that the number of cases in this center is small, and there is selective bias in treatment, it needs to be confirmed by a large number of clinical data.

For IMH patients involving ascending aorta, especially the patients whose initial maximum aortic diameter is ≥ 50 mm or hematoma thickness is ≥ 11 mm, the complication rate and mortality are greater, and the efficacy of intracavitary treatment is better than conservative treatment. It is recommended to actively perform TEVAR. Compared with traditional open surgery, TEVAR will be the beneficial treatment options, especially for those who are unable to tolerate traditional open surgery. When initial maximum aortic diameter is < 50 mm or hematoma thickness is < 11 mm, medical therapy, traditional surgery or TEVAR can be performed. For patients with IMH involving the descending aorta, especially when the maximum aortic diameter is ≥ 40 mm or the hematoma thickness is ≥ 10 mm, the complication rate and mortality are higher. The efficacy of TEVAR is better than conservative treatment. When the initial maximum aortic diameter is < 40 mm and the hematoma thickness is < 10 mm, active drug therapy can be given at the early stage, and regular follow-up review should be performed, for complications such as persistent or recurrent pain, aortic dilation, anatomical progression, terminal organs poor perfusion syndrome, surgery or interventional therapy are advocated. Compared with traditional open surgery, TEVAR has significantly improved surgery-related complications and mortality, and it has a good short-term effect. However, the long-term efficacy needs further follow-up observation. The number of cases in this study is still small, and the follow-up time is short, which needs further confirmation by a large number of clinical data.

## Conclusions

Stanford type A IMH patients when the initial maximum aortic diameter is ≥ 50 mm or the hematoma thickness is ≥ 11 mm and patients with Stanford type B IMH when the initial maximum aortic diameter is ≥ 40 mm or the hematoma thickness is ≥ 10 mm could benefit from the TEVAR.

## Data Availability

Please contact author for data requests.

## References

[1] Vilacosts I, Roman JA (2001). Acute aortic syndrome. Heart.

[2] Mir J, Peihua Z (2011). Clinical Vasopulmonary Surgery 3.

[3] Cronenwett JL, Wayne Johnston K (2014). Rutherford’s Vascular Surgery.

[4] Stanson AW, Kazmier FJ, Hollier LH (1986). Penetrating atherosclerotic ulcers of the thoracic aorta: Natural history and clinicopathologic correlations. Ann. Vasc. Surg..

[5] Eggebrecht H, Plicht B, Kahlert P (2009). Intramural hematoma and penetrating ulcers: Indications to endovascular treatment. Eur. J. Vasc. Endovasc. Surg..

[6] Coady MA, Rizzo JA, Hammond GL (1998). Penetrating ulcer of the thoracic aorta: What is it? How do we recognize it? How do we manage it?. J. Vasc. Surg..

[7] Cho KR, Stanson AW, Potter DD (2004). Penetrating atherosclerotic ulcer of the descending thoracic aorta and arch. J. Thorac. Cardiovasc. Surg..

[8] Nathan DP, Boonn W, Lai E (2012). Presentation, complications, and natural history of penetrating atherosclerotic ulcer disease. J. Vasc. Surg..

[9] Hirst AE, Barbour BH (1958). Dissecting aneurysm with hemopericardium; report of a case with healing. N. Engl. J. Med..

[10] Ganaha F, Miller DC, Sugimoto K (2002). Prognosis of aortic intramural hematoma with and without penetrating atherosclerotic ulcer: A clinical and radiological analysis. Circulation.

[11] Vilacosta I, San Roman JA, Ferreiros J (1997). Natural history and serial morphology of aortic intramural hematoma: A novel variant of aortic dissection. Am. Heart J..

[12] Lissin LW, Vagelos R (2002). Acute aortic syndrome: A case presentation and review of the literature. Vasc. Med..

[13] Erbel R, Alfonso F, Boileau C (2001). Diagnosis and management of aortic dissection. Recommendations of the task force on aortic dissection. Eur. Heart J..

[14] Evangelista A, Dominguez R, Sebastia C (2004). Prognostic value of clinical and morphologic findings in short-term evolution of aortic intramural haematoma. Eur. Heart J..

[15] von Kodolitsch Y, Schwartz AG, Nienaber CA (2000). Clinical prediction of acute aortic dissection. Arch Intern. Med..

[16] Tittle SL, Lynch RJ, Cole PE (2002). Midterm follow-up of penetrating ulcer and intramural hematoma of the aorta. J. Thorac. Cardiovasc. Surg..

[17] Harris KM, Braverman AC, Eagle KA (2012). Acute aortic intramural hematoma: An analysis from the International Registry of acute aortic dissection. Circulation.

[18] Evangelista A, Mukherjee D, Mehta RH (2005). Acute intramural hematoma of the aorta: A mystery in evolution. Circulation.

[19] Daily PO, Trueblood HW, Stinson EB (1970). Management of acute aortic dissection. Ann. Thorac. Surg..

[20] Lizhong S (2005). Diagnosis and treatment of aortic dissection. Chin. J. Surg..

[21] Isselbacher EM, Braunwald E (2002). Diseases of the aorta. Heart Disease.

[22] Robbins RC, McManus RP, Mitchell RS (1993). Management of patients with intramural hematoma of the thoracic aorta. Circulation.

[23] Nienaber CA, von Kodolitsch Y, Petersen B (1995). Intramural hemorrhage of the thoracis aorta: Diagnostic and therapeutic implications. Circulation.

[24] Harris KM, Braverman AC, Gutierrez FR (1997). Transesophageal echocardiographic and clinical features of aortic intramural hematoma. J. Thorac. Cardiovasc. Surg..

[25] Nienaber CA, Sievers HH (2002). Intramural hematoma in acute aortic syndrome: More than one variant of dissection?. Circulation.

[26] Kaji S, Nishigami K, Akasaka T (1999). Prediction of progression or regression of type A aortic intramural hematoma by computed tomography. Circulation.

[27] Song JK, Kim HS, Kang DH (2001). Different clinical features of aortic intramural hematoma versus dissection involving the ascending aorta. J. Am. Coll. Cardiol..

[28] von Kodolitsch Y, Csösz S, Koschyk DH (2003). Intramural hematoma of the aorta: predictors of progression to dissection and rupture. Circulation.

[29] Siminelakis SN, Baikoussis NG, Papadopoulos GS, Beis JP (2009). Axillary artery cannulation for cardiopulmonary bypass during surgery on the ascending aorta and arch. J. Card. Surg..

[30] Kitai T, Kaji S, Yamamuro A (2009). Clinical outcomes of medical therapy and timely operation in initially diagnosed type a aortic intramural hematoma: A 20-year experience. Circulation.

[31] Jianfang L, Huadong L (2014). Interpretation of the guidelines for diagnosis and treatment of Aortic Diseases of the European Society of Cardiology in 2014. Lingnan Cardiovasc. Dis..

[32] Krukenberg E (1920). Contribution to the question of dissecting aneurysm. Beitr Pathol. Anat. Allg. Pathol..

[33] Estrera A, Miller C, Lee TY (2009). Acute type A intramural hematoma: Analysis of current management strategy. Circulation.

[34] Neri E, Capannini G, Carone E (1999). Evolution toward dissection of an intramural hematoma of the ascending aorta. Ann. Thorac. Surg..

[35] Ince H, Nienbar CA (2002). The concept of interventional therapy in acute aortic syndrome. J. Cardiol. Surg..

[36] Dake MD, Miller DC, Semba CP (1994). Transluminal placement of endovascular stent-grafts for the treatment of descending thoracic aortic aneurysms. N. Engl. Med..

[37] Evangelista A, Eagle KA (2009). Is the optimal management of acute type a aortic intramural hematoma evolving?. Circulation.

[38] Tsai TT, Nienaber CA, Eagle KA (2005). Acute aortic syndromes. Circulation.

[39] Nienaber CA, Eagle KA (2003). Aortic dissection: new frontiers in diagnosis and management: Part II: therapeutic management and follow-up. Circulation.

[40] Kaji S, Nishigami K, Akasaka T, Hozumi T, Takagi T, Kawamoto T, Okura H, Shono H, Horibata Y, Honda T, Yoshida K (1999). Prediction of progression or regression of type A aortic intramural hematoma by computed tomography. Circulation.

[41] Song JM, Kim HS, Song JK, Kang DH, Hong MK, Kim JJ, Park SW, Park SJ, Lim TH, Song MG (2003). Usefulness of the initial noninvasive imaging study to predict the adverse outcomes in the medical treatment of acute type A aortic intramural hematoma. Circulation.

